# P14/ARF-Positive Malignant Pleural Mesothelioma: A Phenotype With Distinct Immune Microenvironment

**DOI:** 10.3389/fonc.2021.653497

**Published:** 2021-03-22

**Authors:** Federica Pezzuto, Francesca Lunardi, Luca Vedovelli, Francesco Fortarezza, Loredana Urso, Federica Grosso, Giovanni Luca Ceresoli, Izidor Kern, Gregor Vlacic, Eleonora Faccioli, Marco Schiavon, Dario Gregori, Federico Rea, Giulia Pasello, Fiorella Calabrese

**Affiliations:** ^1^ Department of Cardiac, Thoracic, Vascular Sciences and Public Health, University of Padova, Padova, Italy; ^2^ Department of Surgery, Oncology and Gastroenterology, University of Padova, Padova, Italy; ^3^ Azienda Ospedaliera SS Antonio e Biagio e Cesare Arrigo, Mesothelioma and Rare Cancer Unit, Alessandria, Italy; ^4^ Medical Oncology, Cliniche Humanitas Gavazzeni, Bergamo, Italy; ^5^ Pathology Laboratory, University Clinic Golnik, Golnik, Slovenia; ^6^ Department of Oncology, Medical Oncology 2, Istituto Oncologico Veneto IRCCS, Padova, Italy

**Keywords:** immune microenvironment, MPM, p14/ARF, malignant pleural mesothelioma, tumor microenvironment

## Abstract

**Introduction:**

The CDKN2A gene plays a central role in the pathogenesis of malignant pleural mesothelioma (MPM). The gene encodes for two tumor suppressor proteins, p16/INK4A and p14/ARF, frequently lost in MPM tumors. The exact role of p14/ARF in MPM and overall its correlation with the immune microenvironment is unknown. We aimed to determine whether there is a relationship between p14/ARF expression, tumor morphological features, and the inflammatory tumor microenvironment.

**Methods:**

Diagnostic biopsies from 76 chemo-naive MPMs were evaluated. Pathological assessments of histotype, necrosis, inflammation, grading, and mitosis were performed. We evaluated p14/ARF, PD-L1 (tumor proportion score, TPS), and Ki-67 (percentage) by immunohistochemistry. Inflammatory cell components (CD3+, CD4+, CD8+ T lymphocytes; CD20+ B-lymphocytes; CD68+ and CD163+ macrophages) were quantified as percentages of positive cells, distinguishing between intratumoral and peritumoral areas. The expression of p14/ARF was associated with several clinical and pathological characteristics. A random forest-based machine-learning algorithm (Boruta) was implemented to identify which variables were associated with p14/ARF expression.

**Results:**

p14/ARF was evaluated in 68 patients who had a sufficient number of tumor cells. Strong positivity was detected in 14 patients (21%) (11 epithelioid and 3 biphasic MPMs). At univariate analysis, p14/ARF-positive epithelioid mesotheliomas showed higher nuclear grade (G3) (p = 0.023) and higher PD-L1 expression (≥50%) (p = 0.042). The percentages of CD4 and CD163 in peritumoral areas were respectively higher and lower in p14/ARF positive tumors but did not reach statistical significance with our sample size (both p = 0.066). The Boruta algorithm confirmed the predictive value of PD-L1 percentage for p14/ARF expression in all histotypes.

**Conclusions:**

p14/ARF-positive epithelioid mesotheliomas may mark a more aggressive pathological phenotype (higher nuclear grade and PD-L1 expression). Considering the results regarding the tumor immune microenvironment, p14/ARF-negative tumors seem to have an immune microenvironment less sensitive to immune checkpoint inhibitors, being associated with low PD-L1 and CD4 expression, and high CD163 percentage. The association between p14/ARF-positive MPMs and PD-L1 expression suggests a possible interaction of the two pathways. Confirmation of our preliminary results could be important for patient selection and recruitment in future clinical trials with anticancer immunotherapy.

## Introduction

Malignant pleural mesothelioma (MPM), an occupational disease mainly due to asbestos exposure, is characterized by rapidly progressive and diffusely local growth, late metastases and poor prognosis. Asbestos fibers lead to a protracted immune response and make mesothelioma a neoplasm characterized by a clear immune infiltrate (1). In recent years, the awareness of a strict interaction between tumor cells and tumor microenvironment (TME) (2) have offered new therapeutic opportunities with immunotherapeutic agents (3). One such strategy is based on the treatments targeting the programmed cell death pathway (PD-1/PD-L1) (4). Nevertheless, some limitations persist. They are mainly related to the complexity of the TME structure and the mechanisms of resistance and inhibition, likely associated with the complex genetic profiling of the tumor (5).

The genetics of MPM appear extremely intricate and not completely unraveled. The complexity resides mainly in the variety of genetic aberrations that can occur, the crosstalk between genetics and the microenvironment, and the inter-patient and intra-tumoral variability (6).

In MPM, heterogeneity is indeed also an intrinsic aspect of the neoplasia that has its roots in the histological classification into three major histological types (epithelioid, biphasic, and sarcomatoid) (7), confirmed and emphasized by large-scale molecular profiling studies (8, 9). This is principally true for the epithelioid histotype that includes a wide range of architectural patterns and cytological and stromal features, each of which is supposed to be associated with a different behavior (10). In this complex scenario, the identification of a biomarker makes it possible to stratify the population. When associated with a certain clinical course, this would be useful for diagnostic, prognostic and therapeutic purposes.

Major molecular changes lead to altered expression of the genes involved in oncogenic mechanisms, especially the onco-suppressor genes at 9p21 (INK4) and 22q (NF2) foci (8, 9). The 9p21 locus includes the genes cyclin-dependent kinase inhibitor 2B (CDKN2B), cyclin-dependent kinase inhibitor 2A (CDKN2A) and S-methyl-5’-thioadenosine phosphorylase (MTAP). Frequent involvement of the CDKN2A gene in the pathogenesis of MPM has recently been confirmed (8, 9). The gene encodes for two proteins, p16/INK4A and p14/ARF, both acting as tumor suppressors through the regulation of the cell cycle. p14/ARF is involved in cell cycle regulation, mainly inhibiting MDM2 and promoting p53 function that in turn activates p21. This last protein binds and inactivates cycline-cycline dependent kinase complexes, thus blocking the transition from the G1 to S phases of the cell cycle. The deletion interferes with the p53-MDM2 pathway, leading to accumulation of MDM2 and loss of p53 function with cell cycle deregulation. Even if less common, several p53-independent actions have been attributed to p14/ARF (11). Although p14/ARF deletion or silencing has been found in several solid tumors (12), the role of p14/ARF in the pathogenesis of MPM remains unclear with most evidence dating back to the last decade and being based only on experimental models (13).

In human cell lines, the inactivation/absence of p14/ARF was found to interact with p53 function in case of DNA damage and thus in the apoptotic process (14). p14/ARF (called p19 in mice) alterations were also studied in mouse mesothelioma cells, making mice a feasible model to study molecular features of human MPM (15). Indeed, more recently, p19 status was also studied in *in vivo* experiments, resulting as an important factor for MPM tumorigenesis (16, 17). Interestingly, p14/ARF transfected mesothelioma cells were used to explore new therapeutic approaches, favoring p53 activity and apoptosis, and modulating the cytolytic effects of drugs (18, 19).

Although the role of p14/ARF in tumorigenesis has been widely suggested, its prognostic significance remains unknown (12). Only few studies have clinically evaluated p14/ARF in MPM, achieving inconclusive results (20, 21). In particular the association of p14 with TME has not yet been explored.

Thus, the aim of this study was to investigate p14/ARF expression in MPM and to explore if p14/ARF-positive MPM samples show pathological and immunohistochemical features, with specific focus on immune TME.

## Materials and methods

### Study Population

We retrospectively analyzed biopsies from chemo-naive patients with MPM recruited in three Italian centers and one Slovenian center from 2011 to 2019. Clinical information about patients enrolled in the study were reported and included in an electronic shared data base. Written informed consent was given by all subjects included in the research. The study was approved by the Ethics Committee.

### Histological and Immunohistochemical Evaluation

For histological analyses, tissue samples were fixed in 4% buffered formalin and embedded in paraffin. Each case was classified into epithelioid, biphasic and sarcomatoid, according to the 2015 World Health Organization (WHO) classification (7).

Histological parameters and immunohistochemical evaluations were performed only when biopsies met the following criteria: 1) size adequacy: at least 2 cm^2^/>60% neoplastic cells.

In epithelioid histotypes, the specific architectural pattern was reported and a nuclear grading system (I-II-III) was performed as originally described by Kadota et al. (22). The proliferative index Ki67 antigen was investigated (1:80, Immunotech, clone MIB-1) and expressed as number of positive cells on total cell number.

Necrosis and inflammation were morphologically evaluated both in intratumoral and peritumoral areas and quantified in percentage over the entire tumor surface. The tumor-infiltrating immune cell analysis was based on the guidelines from the International Immuno-Oncology Biomarkers Working Group (23). Briefly, the tumor-infiltrating immune cell analysis was carried out within the borders of the invasive tumor and areas within the tumor. Areas with necrosis, fibrosis/scar or adjacent normal tissue were excluded.

Inflammatory cells were further classified into lymphocytes (B and T) and macrophages (also considering M2 type). The TME characterization was performed by using the following primary antibodies: anti-CD20 (1:200, Dako, clone L26, CD20CY), anti-CD3 (1:200, Leica, clone NCL-L-CD3-565), anti-CD8 (1:200, Dako, clone C8/144B), anti-CD4 (1:200, Dako, clone 4B12), anti-CD68 (1:200, Dako, clone PG-M1), anti-CD163 (1:200, Novocastra, NCL-L3CD163). Immunoreactivity was expressed as percentage of positive cells with respect to the total number of inflammatory cells.

PD-L1 (1:200, cell signaling, clone E1L3N) was evaluated in neoplastic cells and considered to be positive when it was higher than 1%. In a previous study (2) we used an anti-human Ventana PD-L1 rabbit monoclonal primary antibody (clone SP263, pre-diluted, 1,61 μg/mL). After that, we have started to use PD-L1 (clone E1L3N) following a laboratory validation test that demonstrated a similar rate of positivity of the two antibodies. In tumor cells, PD-L1 was scored as Tumor Proportion Score (TPS). p14/ARF (1:100, Santa Cruz, clone 4C6/4ARF) was defined as positive when neoplastic cells showed strong nuclear or both cytoplasmic and nuclear/staining. In all immunostainings negative controls for non-specific binding were included omitting the primary antibodies.

Immunohistochemistry was performed by using the Bond automated system (Leica Bond III, Leica Microsystems Srl, Wetzlar, Germany).

### Statistical Analysis

Data are expressed as medians (interquartile range). Univariate analyses were conducted with the non-parametric Mann-Whitney U or Kruskal-Wallis tests for continuous variables and Fisher’s exact test for categorical variables. Feature selection was implemented using a machine-learning algorithm based on random forest (Boruta). The Boruta algorithm aims to identify all the relevant predictors that impact the outcome of interest (in our case, being in the p14/ARF+ or p14/ARF- group). It implements a random forest on an augmented set of covariates. Additional covariates, called shadow variables, are copies of the original ones obtained by permuting the observations and thus removing any possible association with the outcome. For each explanatory variable, an importance measure is computed, i.e., a Z score, which is the average improvement in the predictive performance of the random forest with the considered explanatory variable divided by its standard deviation. The obtained important predictors are those that show a Z score higher than the one observed for the variable with the maximum Z score among the shadow variables. The procedure is repeated until an importance measure is assigned to each predictor or until the maximum number of random forests is reached. The Boruta R package (24) was used for the analysis. The Boruta feature selection is a heuristic algorithm of machine learning that was used to highlight the most important variables that were able to distinguish the p14/ARF positiveness. The algorithm is based on the random forest algorithm and permits to compare variables randomizing both data and variables in order to obtain independent decision trees that are finally used to produce a decision about the most influencing, group-diving features. Survival was evaluated using Kaplan-Meyer curves and Log-rank test. Moreover, survival curves were also compared with the non-parametric restricted mean survival time as a summary measure of the survival time distribution adjusted for age, sex, and chemotherapy and/or surgery treatments at 6 and 12 months with the survival and survRM2 R packages. Graphs were made with the ggstatsplot R package. R (v. 4.0.3) was used for the analysis (25).

## Results

### Patients

Seventy-six chemo naive MPM patients were enrolled in the study. p14/ARF was evaluable in 68 (89%) MPM samples containing a sufficient number of tumor cells. Most patients were males (51/68, 75%), with a median age of 72 (61.8-76; Q1-Q3) years. A positive history of asbestos exposure was obtained from 62%. According to the Eastern Cooperative Oncology Group (ECOG), performance status (PS) was 0 in 32%, 1 in 62% and 2 in 6%. Median survival was 9.3 (5.5-12.9; Q1-Q3) months. The main clinical data are summarized in **Table 1**.

**Table 1:** Main clinical features of patients affected by malignant pleural mesothelioma.

	p14/ARF positive (n = 14)	p14/ARF negative(n = 54)	TOTAL(n = 68)
**Sex [F:M]**	5:9	12:42	17:51
**Age [yrs, median (Q1-Q3)]**	64 (61.2-73.5)	72.5 (63.0-76.0)	72.0 (61.8-76.0)
**Overall Survival [months, median (Q1-Q3)]**	9.3 (5.8-11.7)	9.8 (5.6-14.4)	9.3 (5.5-12.9)
**Asbestos Exposure [%]**			
**Yes**	63	57	62
** No**	28	43	32
**Not available**	9	0	6
**ECOG PS [%]**			
**0**	22	35	32
** 1**	64	61	62
** 2**	14	4	6
**EORTC PrS [median (Q1-Q3)]**	1.75 (1.5-1.8)	1.67 (1.15-1.82)	1.72 (1.15-1.82)
**Surgery [%]**			
** Yes**	0	100	22
** No**	26	74	78
**Chemotherapy [%]**			
**Yes**	22	78	75
** No**	43	57	10
** Not available**	0	100	15
**Multimodal Treatment [%]**			
**Yes**	0	100	22
** No**	26	74	78

ECOG, Eastern Cooperative Oncology Group; PS, performance status; EORTC, European Organization for Research and Treatment of Cancer; PrS, prognostic score.

### Morphological Characteristics, TME/PD-L1 and p14/ARF Expression

Forty-seven cases were epithelioid (69%), 17 biphasic (25%) and 4 sarcomatoid (6%). Epithelioid histotype displayed a solid prevalent growth pattern (25 out of 47, 53%). The majority of epithelioid MPM had mild/moderate nuclear grading (13 cases with G1, 21 G2) while 28% (13 G3) showed severe nuclear grading.

Necrosis was detected in 48 cases (71%) with a median percentage of 10.0 (5.0-18.5; Q1-Q3). Loss of nuclear immunoreactivity of p14/ARF was evident in 54 cases (79%). Strong nuclear expression was mainly detected in epithelioid mesotheliomas (11 out of 14, 79%). In 79% of positive cases cytoplasmic immunoreactivity was detected in addition to the nuclear staining.

TME was evaluable in 68 cases. Inflammation showed a median of 10.0 (5.0-17.5; Q1-Q3), higher in epithelioid than non-epithelioid (14.0 vs 10.0). PD-L1 was expressed in 37 (54%) cases (23 epithelioid and 14 non-epithelioid: 12 biphasic and 2 sarcomatoid). PD-L1 was strongly positive with a TPS ≥ 50% in 17 (25%) cases.

### Relationship Between p14/ARF and Clinicopathological Data

**Figure 1:**
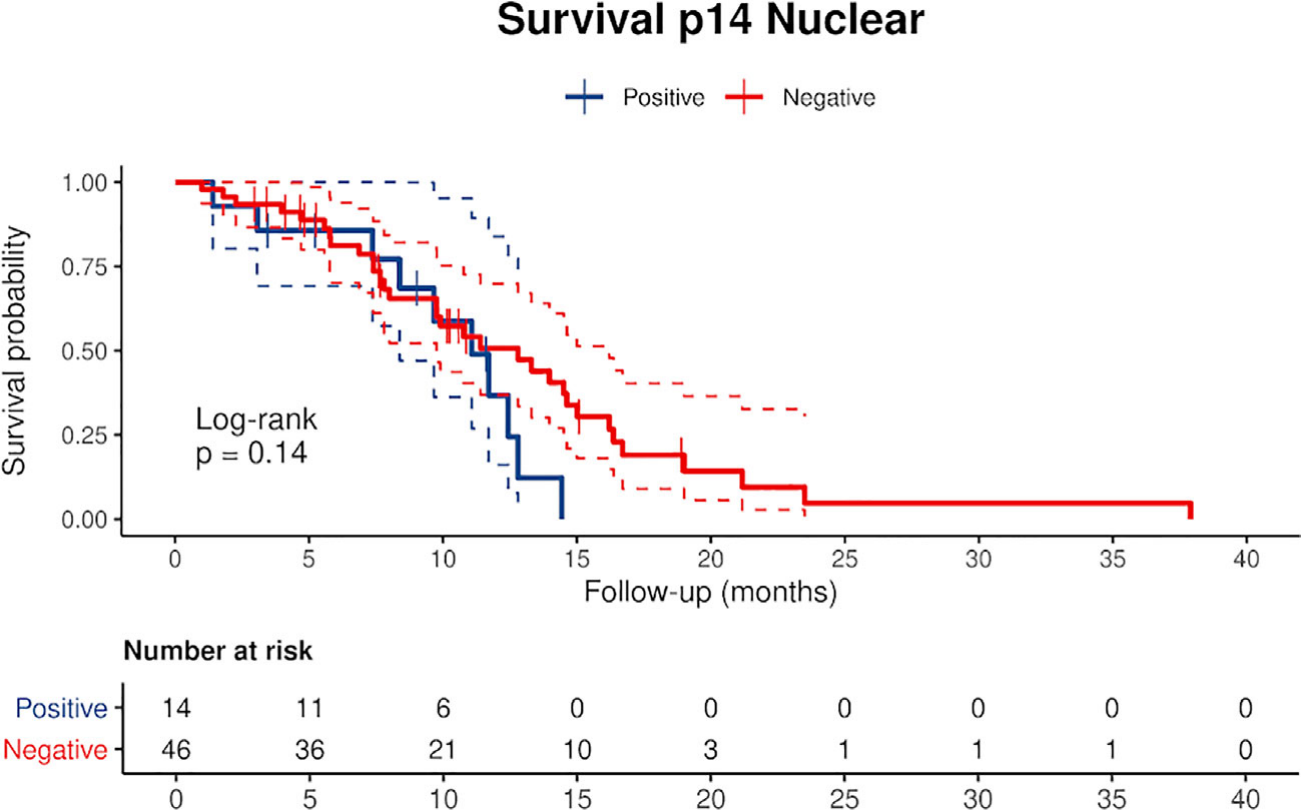
Kaplan Meier curves of p14/ARF positive and negative cases showing a lower survival rate in patients with p14/ARF expression. Censored patients are depicted as crosses intersecting the curve.

**Figure 2:**
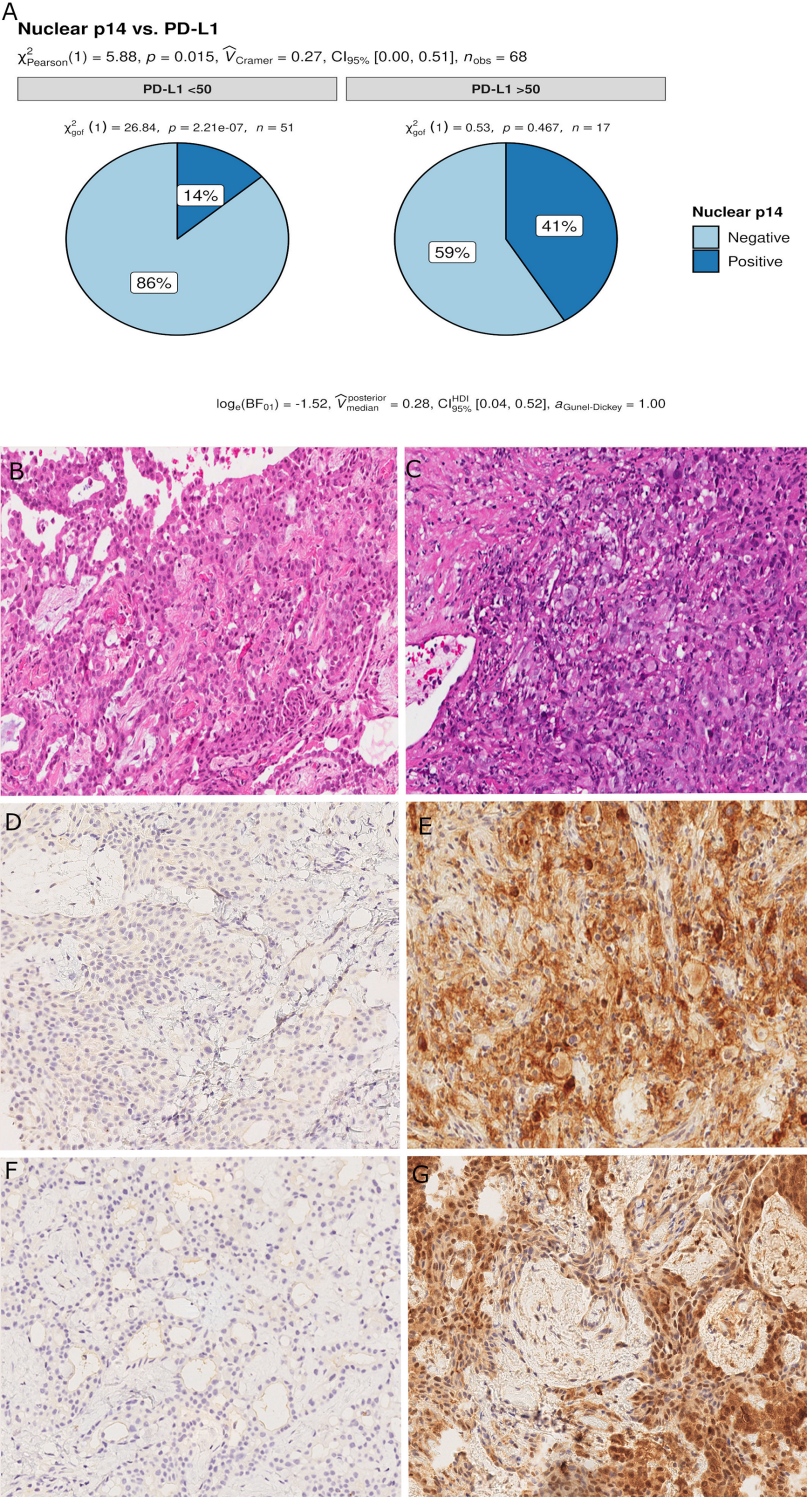
Pie chart of PD-L1 expression in p14/ARF-positive and negative samples. A higher percentage of PD-L1≥50% was noted in p14/ARF-positive samples than in p14/ARF-negative MPMs **(A)**. Panel figures of two representative cases of p14/ARF-negative **(B, D, F)** and p14/ARF-positive MPM **(C, E, G)**. **(B)** Histology showing trabecular pattern of MPM (hematoxylin and eosin, original magnification x 200). **(D)** Immunohistochemistry for PD-L1: TPS<1% (immunohistochemistry, original magnification x 200). **(F)** Immunohistochemistry for p14/ARF: negative (immunohistochemistry, original magnification x 200). **(C)** Histology showing prevalent solid pattern of MPM (hematoxylin and eosin, original magnification x 200). **(E)** Immunohistochemistry for PD-L1: TPS≥50% (80%) (immunohistochemistry, original magnification x 200). **(G)** Immunohistochemistry for p14/ARF: positive (immunohistochemistry, original magnification x 200).

**Figure 3:**
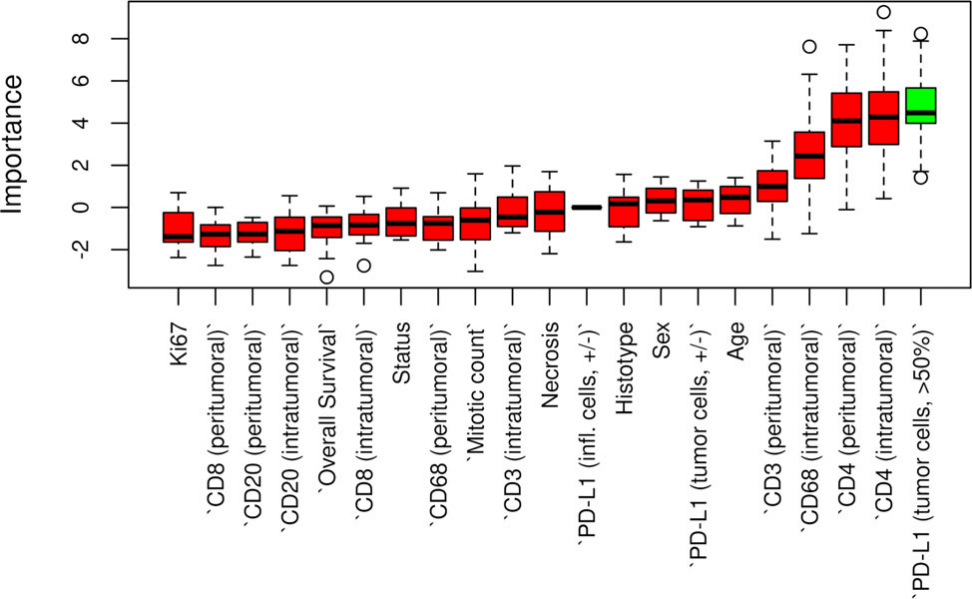
Boruta feature selection showing predictive significance for PD-L1 expression in p14/ARF positivity. Unfilled circles indicating outliers.

**Figure 4:**
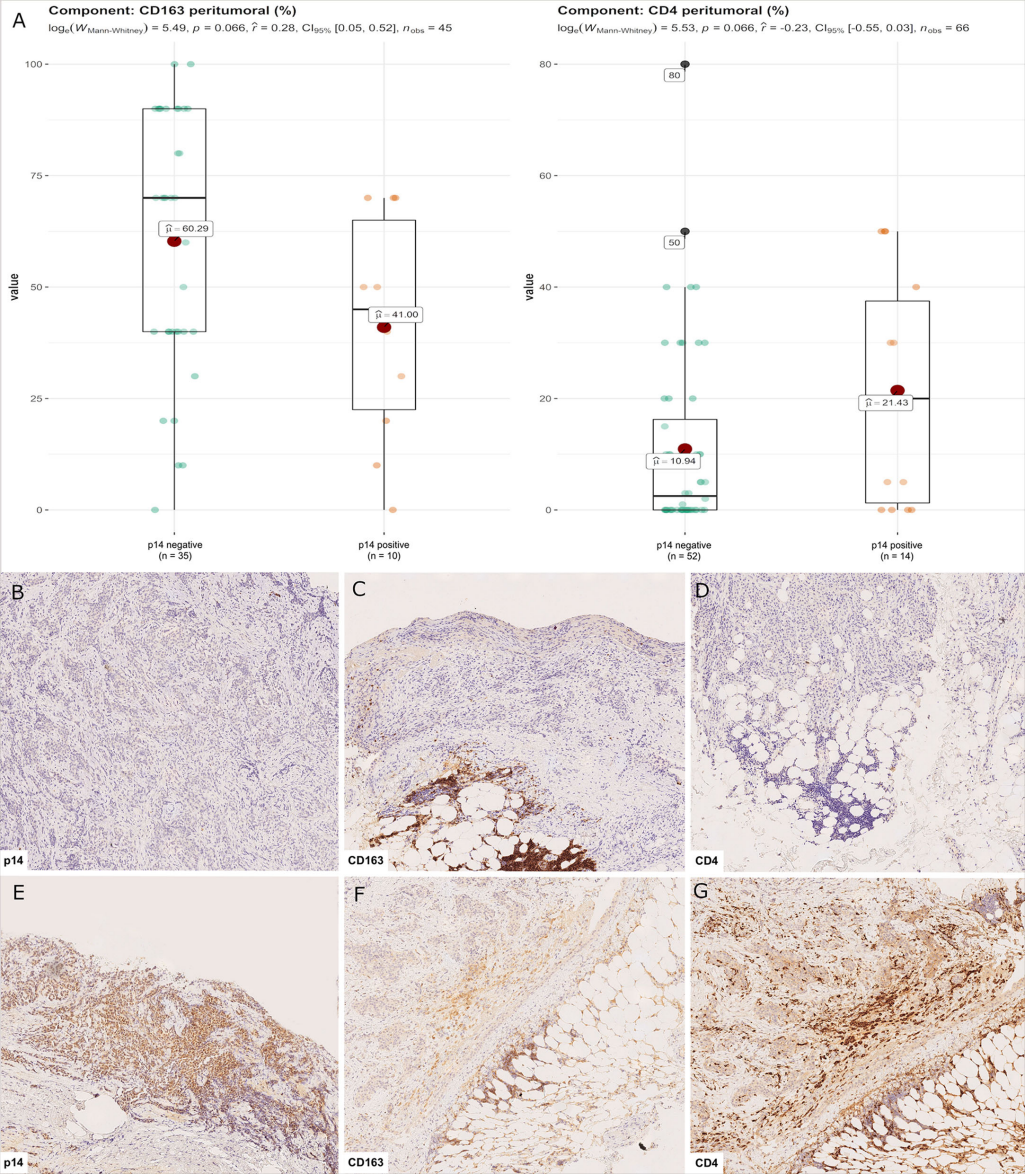
CD4+ and CD163+ distribution in peritumoral areas. T helper lymphocytes and M2 macrophages were respectively higher and lower in p14/ARF-positive tumors than in negative samples **(A)**. Panel figures of two representative cases of p14/ARF-negative **(B–D)** and p14/ARF-positive MPM **(E–G)**. **(B)** Immunohistochemistry for p14/ARF: negative (immunohistochemistry, original magnification x 70). **(C)** Immunohistochemistry for CD163 showing a high percentage in peritumoral areas (immunohistochemistry, original magnification x 70). **(D)** Immunohistochemistry for CD4 showing a low percentage in peritumoral areas (immunohistochemistry, original magnification x 70). **(E)** Immunohistochemistry for p14/ARF: positive (immunohistochemistry, original magnification x 70). **(F)** Immunohistochemistry for CD163 showing a low percentage in peritumoral areas (immunohistochemistry, original magnification x 70). **(G)** Immunohistochemistry for CD4 showing a high percentage in peritumoral areas (immunohistochemistry, original magnification x 70).

Censored patients were 4 in the p14-positive group and 14 in the negative group. While there was no significant difference in median overall survival (9.3 vs 9.8 months), it was noted that more patients with lack of p14/ARF expression showed survival beyond 10 months. Including chemotherapy, surgery, or their combination as covariates in the survival analysis, the results did not change (**Figure 1**). Restricted mean survival time at 6 and 12 months showed no differences between the two arms adjusted for age and sex: 6 months estimate -0.24 (95% CI -1.02; 0.53, p = 0.536): 12 months estimate -0.34 (95%CI -2.10; 1.71, p = 0.742). p14/ARF expression was correlated with histotype, necrosis, inflammation, all inflammatory cell subtypes and PD-L1 values; at univariate analysis, a significant association was achieved between p14/ARF positivity and high PD-L1 expression in tumor cells (p = 0.015) (**Figure 2**). This strict correlation was also confirmed by the Boruta feature selection that, among all variables evaluated, showed a predictive significance for this parameter (**Figure 3**). Moreover, the percentages of CD4 and CD163 in peritumoral areas were respectively higher and lower (but not significantly) in p14/ARF positive tumors (both p = 0.066) (**Figure 4**). The results are summarized in **Table 2**.

**Table 2:** Main histological features of malignant pleural mesothelioma distinguishing p14/ARF positive and negative cases.

	p14/ARF positive (n = 14)	p14/ARF negative (n = 54)	TOTAL (n = 68)	p-value
**Histotype (n, %)**				
**Epithelioid**	11 (23%)	36 (77%)	47 (69%)	0.339
**Biphasic**	3 (18%)	14 (82%)	17 (25%)	
**Sarcomatoid**	0	4 (100%)	4 (6%)	
**Necrosis [%, median (Q1-Q3)]**	15.0 (10.0–15.0)	7.0 (5.0–19.0)	10.0 (5.0–18.5)	0.229
**Mitoses [n/mm^2^, median (Q1-Q3)]**	4.0 (3.0–4.8)	3.0 (2.0–5.0)	3.0 (2.0–5.0)	0.478
**Proliferative index [%, median (Q1-Q3)]**	40.0 (25.0, 65.0)	30.0 (20.0, 52.5)	30.0 (20.0–57.5)	0.553
**PD-L1 tumor cells**				
**<50%**	7 (50%)	44 (81.5%)	51 (75%)	0.015*
**≥50%**	7 (50%)	10 (18.5%)	17 (25%)	
**CD8 [%, median (Q1-Q3)]**				
**Peritumoral**	20.0 (15.0–30.0)	20.0 (8.5–30.0)	20.0 (10.0–30.0)	0.614
**Intratumoral**	20.0 (10.0–30.0)	20.0 (5.0–50.0)	20.0 (6.2–50.0)	0.910
**CD4 [%, median (Q1-Q3)]**				
**Peritumoral**	20.0 (1.2–37.5)	2.5 (0.0–16.2)	5.0 (0.0–20.0)	0.066
**Intratumoral**	5.0 (0.5–10.0)	0.0 (0.0–10.0)	0.0 (0.0–10.0)	0.174
**CD20 [%, median (Q1-Q3)]**				
**Peritumoral**	25.0 (15.0–40.0)	20.0 (5.0–40.0)	20.0 (6.2–40.0)	0.626
**Intratumoral**	0.0 (0.0–4.0)	0.0 (0.0–5.0)	0.0 (0.0–5.0)	0.841
**CD20 [%, median (Q1-Q3)]**				
**Peritumoral**	25.0 (15.0–40.0)	20.0 (5.0–40.0)	20.0 (6.2–40.0)	0.626
**Intratumoral**	0.0 (0.0–4.0)	0.0 (0.0–5.0)	0.0 (0.0–5.0)	0.841
**CD3 [%, median (Q1-Q3)]**				
**Peritumoral**	40.0 (16.2–57.5)	30.0 (10.0–40.0)	30.0 (13.8–50.0)	0.266
**Intratumoral**	17.5 (10.0–46.2)	20.0 (5.0–40.0)	20.0 (5.0–, 42.5)	0.813
**CD68 [%, median (Q1-Q3)]**				
**Peritumoral**	30.0 (25.0–47.5)	30.0 (15.0–40.0)	30.0 (20.0–40.0)	0.250
**Intratumoral**	27.5 (20.0–40.0)	40.0 (20.0–60.0)	40.0 (20.0–, 60.0)	0.129
**CD163 [%, median (Q1-Q3)]**				
**Peritumoral**	45.0 (22.5–65.0)	70.0 (40.0–90.0)	60.0 (40.0–80.0)	0.066
**Intratumoral**	35.0 (22.5–47.5)	40.0 (30.0–80.0)	40.0 (30.0–70.0)	0.192

* and bold for statistical significance.

**Figure 5:**
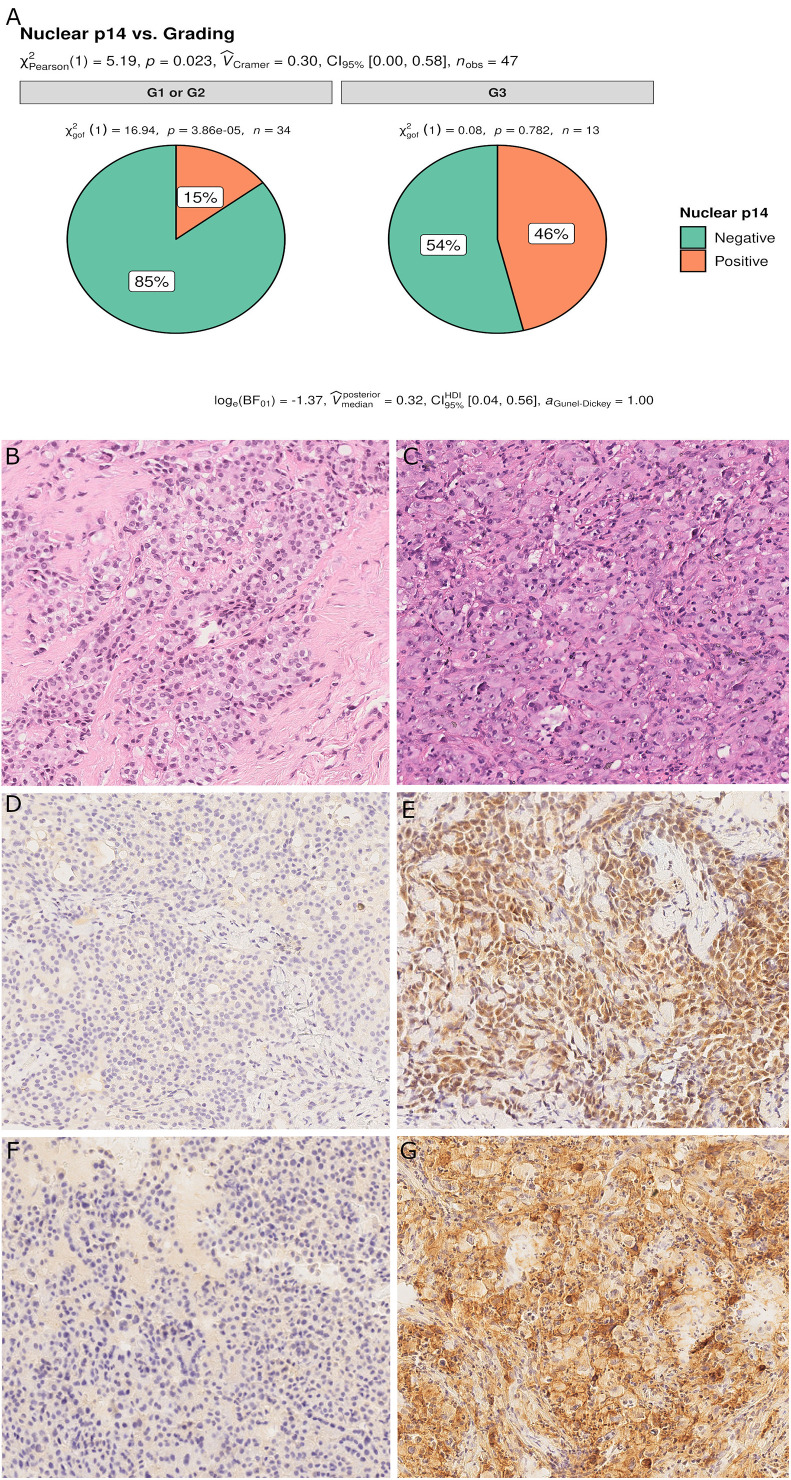
Pie chart of nuclear grading in p14/ARF-positive and negative samples. p14/ARF-positive samples showed higher nuclear grade (G3) than p14/ARF-negative MPM **(A)**. Panel figures of two representative cases of p14/ARF-negative **(B, D, F)** and p14/ARF positive MPM **(C, E, G)**. **(B)** Histology showing an epithelioid MPM with low nuclear grading (G2 sec. Kadota et al.) (hematoxylin and eosin, original magnification x 200). **(D)** Immunohistochemistry for p14/ARF showing complete negative immunostaining (immunohistochemistry, original magnification x 200). **(F)** Immunohistochemistry for PD-L1: TPS<1% (immunohistochemistry, original magnification x 200). **(C)** Histology showing an epithelioid MPM with high nuclear grading (G3 sec. Kadota et al.) (hematoxylin and eosin, original magnification x 200). **(E)** Immunohistochemistry for p14/ARF showing strong nuclear and cytoplasmic immunostaining in most tumor cells (immunohistochemistry, original magnification x 200). **(G)** Immunohistochemistry for PD-L1: TPS≥50% (immunohistochemistry, original magnification x 200).

For epithelioid MPM, 13 cases (28%) had a high nuclear grade (G3). p14/ARF expression was significantly associated with a higher nuclear grade (p = 0.023) (**Figure 5**). As would be expected since almost all p14/ARF positive MPM samples were epithelioid, a high PD-L1 expression was associated with p14/ARF expression (p = 0.042). No other differences were found between the two groups.

**Figure 6:**
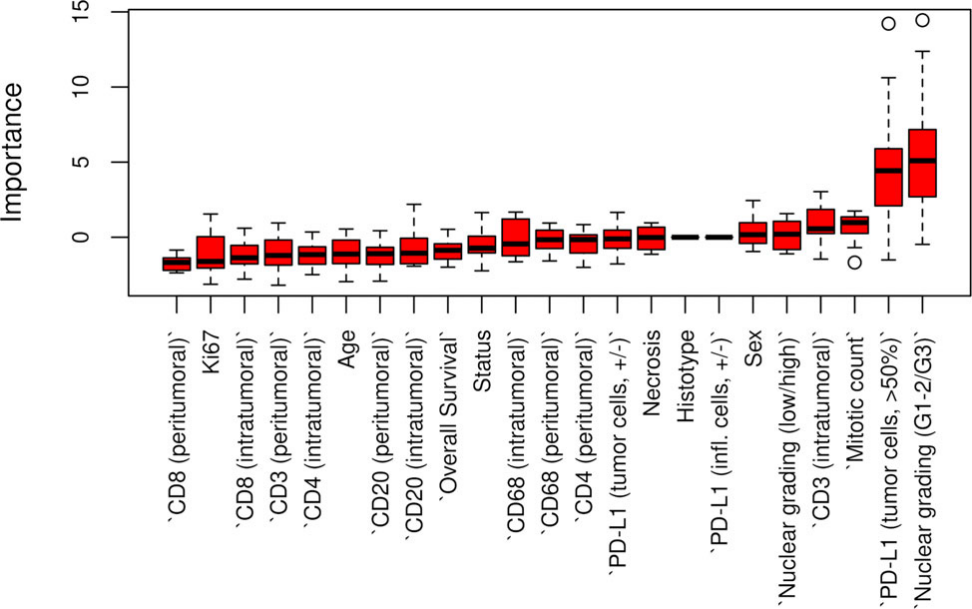
Boruta feature selection showing the highest importance of nuclear grade and PD-L1 expression among all variables in determining p14/ARF status. Unfilled circles indicating outliers.

The Boruta feature selection showed that nuclear grade and PD-L1 expression were the two most important variables in determining the p14/ARF status, although significance was not achieved (**Figure 6**).

## Discussion

In the present study, we detected tissue expression of p14/ARF in only 21% of MPM, mainly epithelioid histotype (79%) thus confirming previous studies on MPM that showed a more frequent loss of the CDKN2A gene (10).

An interesting finding of our study was that MPM with p14/ARF expression showed a higher nuclear grading, more extensive necrosis (this parameter also did not reach statistical significance) and a higher PD-L1 TPS value. All these findings seem to characterize more aggressive pathological forms. The CDKN2A locus expresses two partially overlapping transcripts that encode two distinct proteins, namely p14/ARF and p16/INK4a, which present no sequence identity. While several experimental studies showed that both proteins are potent tumor suppressors, the importance of p14/ARF alterations in several human cancers remains unclear (23). Novel data collected in recent years have challenged the traditional and established role of this protein as a tumor suppressor. In particular it has been demonstrated that several tumors retaining p14/ARF expression evolve to metastatic and invasive phenotypes and in humans are associated with a poor prognosis as detected in a subset of our cases (26, 27).

There are only two previous clinical studies that investigated the expression of p14/ARF in MPM which used molecular analysis or immunostaining (20, 21) that did not however allow for univocal interpretation of clinical correlations, particularly the correlation with survival. In our study, Kaplan-Meier curves separated patients with low expression from patients with high expression (**Figure 1**). Curves seem to diverge around 10 months but the different and small numbers of the two groups did not allow a clear evaluation of the two profiles. Restricted mean survival time at 6 and 12 months did not show evidence of different survival in the two groups.

Our findings are apparently in contradiction with those obtained in the study by Walter et al. who reported elevated p14/ARF expression correlated with prolonged survival. However, the authors evaluated p14/ARF using a different methodology: by qPCR.

mRNA is usually translated into protein under the assumption that there is some sort of correlation between level of mRNA and level of protein. However, there may be reasons for the typically poor correlations between mRNA and protein levels, and these reasons may not be mutually exclusive. In particular, low expression of mRNA and protein abundance may be related to the fact that proteins have very different half-lives as the result of varied protein syntheses and degradations. Indeed, protein turnover can vary significantly depending on a number of different conditions. Future more in-depth studies are needed to investigate both *in vitro* and *in vivo* post-transcriptional p14/ARF activity.

In contrast with the exclusively nucleolar localization of p14/ARF observed in most *in vitro* models and in several tumors, abnormal p14/ARF nucleo-cytoplasmic accumulation was found in the majority of our cases. This has been observed in other tumors (27, 28).

It does not seem likely that this is a consequence of nonspecific staining, as a distinct nucleolar signal has been obtained with the same antibody and under the same conditions in other tumor tissues (28). The presence of other nuclear markers (WT1; Ki67) without cytoplasmic spreading demonstrated that our observations are not an artifact related to loss of nuclear integrity. We ignore the significance of nucleocytoplasmic staining—it would be expected that under conditions of massive p14/ARF induction the nucleoplasmic fraction would become detectable as was in many of our cases.

In the present study it was demonstrated that there was an association between p14/ARF and PD-L1 expression in mesothelial cells, which may be explained by the response of neoplastic cells to immune attack. Several experimental and clinical studies have demonstrated that p14/ARF is a critical modulator of the inflammatory response although its exact role in the complex regulation of an inflammatory tumor microenvironment is still unclear. Inflammatory tumor microenvironments contribute to the carcinogenesis and progression of several types of solid and hematologic cancers. PDL-1 is an immune modulatory molecule in cancer cells that inhibits cytotoxic T cell activity (29) thereby enabling tumor growth (30). Chronic inflammation due to inhaled asbestos in the pleura and/or into the lung has been thought to play a major role in MPM pathogenesis. Recently, the use of immune checkpoint inhibitor (ICI) as single agents or in combination in previously treated and naïve patients has been shown to potentially prolong MPM patient survival even if the value of single agent checkpoint inhibitors is rather limited yielding overall response rates with immunotherapy around 30% (31). Even if more recently the Checkmate-743 study demonstrated a significant improvement in overall survival for the combination of nivolumab and ipilimumab (32), some issues about the expected response and the acceptable toxicity need to be addressed. While the clinical efficacy of ICI has been claimed to correlate with a high tumor mutational burden as in melanoma or NSCLC patients, mesothelioma has consistently been demonstrated to harbor a low mutation burden (10). This may explain the low sensitivity to the ICI targeting PD-1/PDL-1; however, the possible influence of some genes that could have the same efficacy of ICI in MPM patients is also important. A role for p14/ARF in the innate immune response has been previously demonstrated, although the underlying mechanisms are unclear (33–35). The mechanisms include the modulation of angiogenesis, matrix remodeling, and immune suppression (36). In vitro and *in vivo* models have demonstrated a significant influence of p19/ARF in regulating the plasticity and polarization of macrophages. Mice lacking the p19/ARF gene showed a balance with prevalent M2 macrophage phenotypes characterized by the expression of a series of chemokines, cytokines, and proteases that promote immunosuppression (33–36). This seems to be in line with our results as ARF-negative MPM showed higher levels of CD163 than positive MPM.

It could be speculated that p14/ARF and PD-L1 positive mesothelioma characterizes tumor phenotypes with an inflammatory TME that might be more sensitive to the ICI treatment.

There were some limitations of the present study. First, due to the small sample size, the study could be underpowered. Nonetheless, we implemented robust and reliable methods to limit the probability of getting significant results by chance. Moreover, the strength of our research is that all patients included in the current study were chemo-naive and that it overcomes an important selection bias that could influence the evaluation of TME and PD-L1. Concerning p14/ARF immunostaining, there is no standardization for defining p14/ARF positivity. In this regard, further studies are required considering that the absence of a cut-off value may influence the detection rate of positive samples. This is also true for the evaluation of PD-L1 that is not standardized in MM, neither in terms of clones nor type of expression evaluation and threshold.

Finally, although a significant association between PD-L1 and p14/ARF expression was identified, the molecular substrate that influences this association remains unknown. Nonetheless, this study may serve as an important starting point for future genetic and functional studies.

In conclusion, we found an association between p14/ARF-positive MPM and a peculiar MPM phenotype, characterized by a higher nuclear grading, PD-L1 expression, and peculiar inflammatory TME (high number of peritumoral CD4 T-lymphocyte and low number of M2 macrophages). The significant correlation between p14/ARF positive MPM and PD-L1 expression suggests a possible interaction of the two pathways.

There is an urgent need for biomarkers to select the optimal candidates for immunotherapy among MPM patients moreover in terms of efficacy to the ICI treatment. The confirmation of our preliminary results could be useful for patient selection and recruitment in future clinical trials with anticancer immunotherapy to optimize the benefit and the effectiveness of these drugs in MPM.

## Data Availability Statement

The raw data supporting the conclusions of this article will be made available by the authors, without undue reservation.

## Ethics Statement

The studies involving human participants were reviewed and approved by Istituto Oncologico Veneto IRCCS, Padova, Italy. The patients/participants provided their written informed consent to participate in this study.

## Author Contributions

Conception/design: FP, FL, GP, and FC. Data acquisition: FP, FF, GP, FG, GC, IK, GV, MS, and LU. Data analysis/interpretation: LV and DG. Drafting: FP, FL, GP, LV, and FC. Critical revision: FC, GP, DG, and FR. All authors contributed to the article and approved the submitted version.

## Funding

This work was supported by the University of Padova (BIRD197018/19: Influence of tumor microenvironment, metabolic interaction and telomere dysfunction in malignant pleural mesothelioma: translational and preclinical study).

## Conflict of Interest

The authors declare that the research was conducted in the absence of any commercial or financial relationships that could be construed as a potential conflict of interest.
